# Routine Echocardiography and Artificial Intelligence Solutions

**DOI:** 10.3389/fcvm.2021.648877

**Published:** 2021-02-23

**Authors:** Mark J. Schuuring, Ivana Išgum, Bernard Cosyns, Steven A. J. Chamuleau, Berto J. Bouma

**Affiliations:** ^1^Amsterdam University Medical Centers -Location Academic Medical Center, Department of Cardiology, University of Amsterdam, Amsterdam, Netherlands; ^2^Amsterdam University Medical Centers -Location Academic Medical Center, Department of Biomedical Engineering and Physics, University of Amsterdam, Amsterdam, Netherlands; ^3^Amsterdam University Medical Centers -Location Academic Medical Center, Department of Radiology and Nuclear Medicine, University of Amsterdam, Amsterdam, Netherlands; ^4^Amsterdam Cardiovascular Sciences, Amsterdam University Medical Centers -Location Academic Medical Center, University of Amsterdam, Amsterdam, Netherlands; ^5^Department of Cardiology, University Hospital Brussel, Brussels, Belgium

**Keywords:** echocardiography, cardiac imaging, artificial intelligence, image analysis, diagnosis, prediction

## Abstract

**Introduction:** Echocardiography is widely used because of its portability, high temporal resolution, absence of radiation, and due to the low-costs. Over the past years, echocardiography has been recommended by the European Society of Cardiology in most cardiac diseases for both diagnostic and prognostic purposes. These recommendations have led to an increase in number of performed studies each requiring diligent processing and reviewing. The standard work pattern of image analysis including quantification and reporting has become highly resource intensive and time consuming. Existence of a large number of datasets with digital echocardiography images and recent advent of AI technology have created an environment in which artificial intelligence (AI) solutions can be developed successfully to automate current manual workflow.

**Methods and Results:** We report on published AI solutions for echocardiography analysis on methods' performance, characteristics of the used data and imaged population. Contemporary AI applications are available for automation and advent in the image acquisition, analysis, reporting and education. AI solutions have been developed for both diagnostic and predictive tasks in echocardiography. Left ventricular function assessment and quantification have been most often performed. Performance of automated image view classification, image quality enhancement, cardiac function assessment, disease classification, and cardiac event prediction was overall good but most studies lack external evaluation.

**Conclusion:** Contemporary AI solutions for image acquisition, analysis, reporting and education are developed for relevant tasks with promising performance. In the future major benefit of AI in echocardiography is expected from improvements in automated analysis and interpretation to reduce workload and improve clinical outcome. Some of the challenges have yet to be overcome, however, none of them are insurmountable.

## Introduction

Echocardiography is the most commonly performed non-invasive cardiac procedure. It is the recommended imaging modality for most cardiac diseases for diagnostic and prognostic purposes by the European Society of Cardiology (1–7). Echocardiography has unique characteristics such as portability, high temporal resolution, absence of ionizing radiation and low-costs. Precise and reliable echocardiographic assessment is prerequisite for high-quality clinical decision-making (8).

Analysis of echocardiography is associated with numerous challenges. Given that it is recommended as first-line diagnostic tool, an ongoing growing worldwide challenge is to process millions of echocardiography clips and images obtained daily. The increasing workload in all echocardiographic laboratories and varying image quality makes thorough and timely interpretation challenging. Technicians acquire the clips and images, perform manual measurements, write the draft report, which is followed by approval of cardiologists making the total process rather complex, resource intensive and time consuming (9). Cardiologists in private practices and those in small hospitals often do not have technicians available. Hence, they are often overloaded with routine tasks inherent to echocardiographic exams and sometimes miss very specialized expertise. Moreover, it also takes years of education and experience for a technician or cardiologist to become an expert in detecting perceptual cues in echocardiography clips and automatically integrating this information into a clinical differentiation based upon pattern recognition without overt statistical reasoning. Echocardiography is also increasing in complexity, particularly strain imaging and three dimensional (3D) analysis (10). Furthermore, volume of exams is rising due to new diagnostic assessments and therapeutic options leading to a further increase in expert workload (11–13).

**Figure 1:**
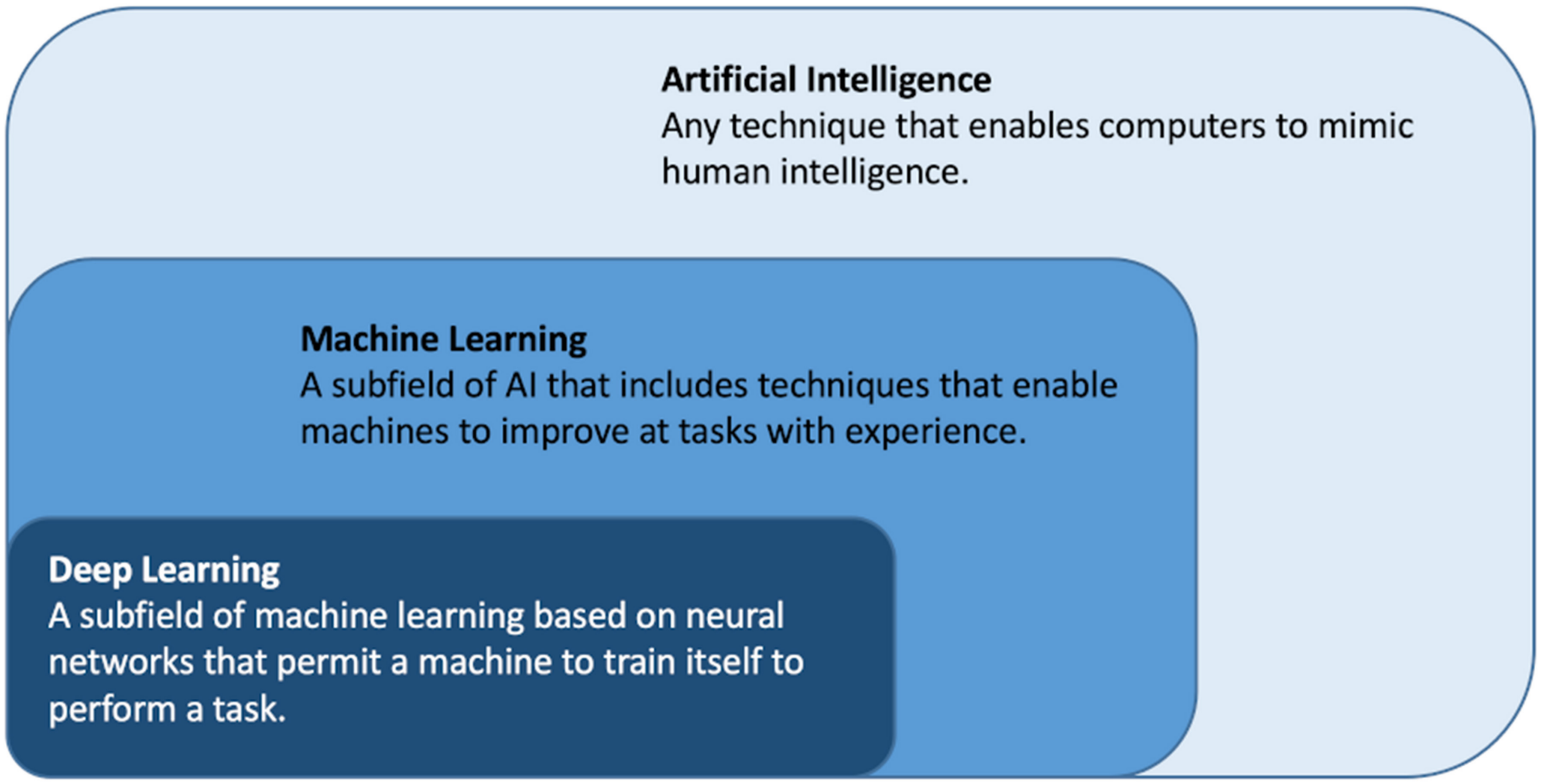
Artificial intelligence, machine learning and deep learning.

Artificial intelligence (AI) is a rapidly emerging field and refers to the broad concept of simulating human logic and intelligence and covers any algorithm or model executed by a computer that mimics human intelligence, see Figure 1 (14–16). Machine learning (ML) is a subfield of AI where the algorithms learn to perform a task based on expert engineered characteristics describing the data (17). Deep learning (DL) is a subfield of ML where the algorithms learn directly from the data themselves circumventing the feature engineering. ML and DL techniques are described in detail elsewhere (18). Handling high complexity, high dimensional data; particularly time series and machine generated data is a strength of many ML and DL algorithm (18).

During development of AI methods, the data sets are partitioned into training, validation and test sets. The training set often encompasses the bulk of all available data and together with a smaller validation set, it is used for the development of the AI method (19). The hold-out test set is used to evaluate overall performance. To evaluate generalizability of the AI solution with respect to e.g., image acquisition or imaged population diverse datasets are required. Application of these approaches for analysis of echocardiography clips and images creates opportunities for automation of expert analysis to advent the acquisition and analysis and thereby improve the clinical workflow (18, 20, 21).

## Routine Echocardiography and Artificial Intelligence Solutions

**Figure 2:**
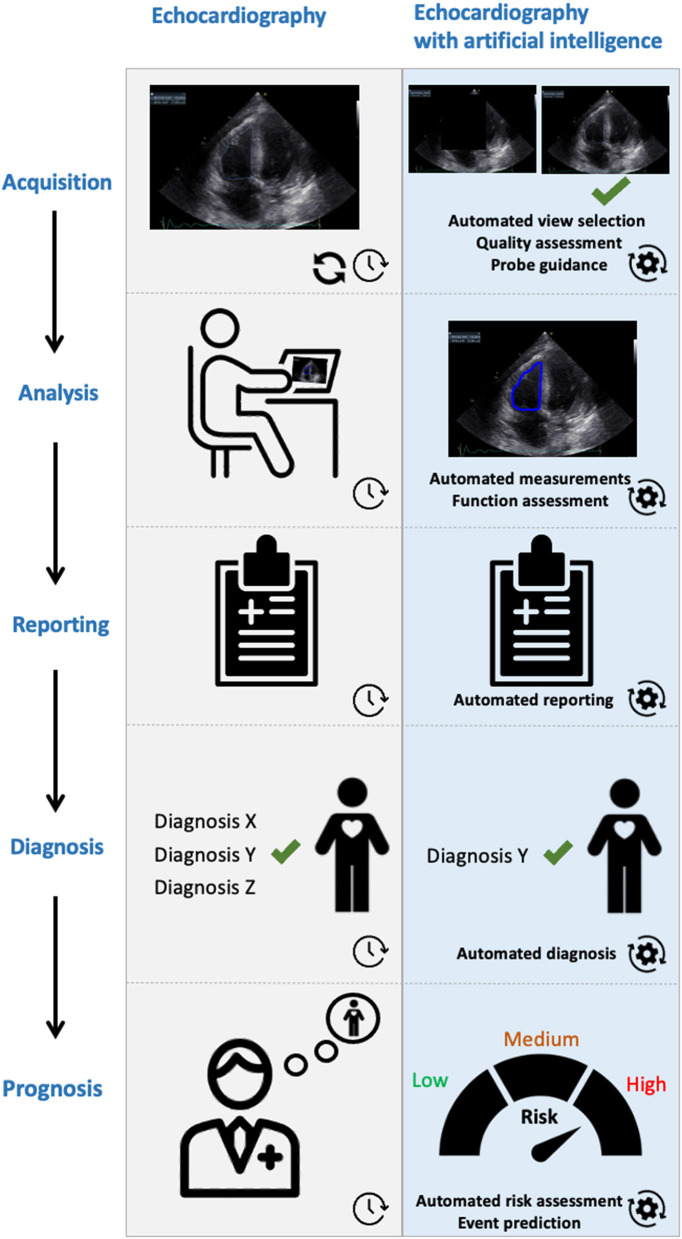
Graphical abstract on future expectations. AI will support doctors, not replace them.

Here, we provide an overview of the AI methods developed for analysis of routine echocardiography. Advanced solutions like those for fusion imaging and role of 3D augmented virtual reality are beyond the scope of this paper. Thus far developed methods are mostly focusing on automated image view selection and image segmentation. One of the important steps in echocardiography is selecting the best view for the subsequent analysis. This can be challenging, and hence time consuming, particularly for inexperienced operators. Subsequently, we discuss AI solutions for diagnosis and prognostication in various diseases. A graphical abstract is shown in Figure 2.

### Automated Image View Classification

Automated view classification can help standardizing views and measurements in echocardiography exams. Moreover, automated view classification can help non-experts to start learning understanding and using echocardiography (12). During training, automatic tools classifying cardiac views could recognize off-axis acquisition and incorrect views and provide guidance on how to move the probe in order to obtain the correct diagnostic images. Nowadays, the acquisition of ultrasound images is still usually performed in two-dimensional (2D) mode (22). Khamis et al. developed a method exploiting supervised dictionary learning for classification of apical two-chamber (AP2CH), four-chamber (AP4CH), and long-axis (AL) views using clips of 103 patients. The evaluation demonstrated a classification accuracy of more than 91% in all views (23). Gao et al. performed viewpoint classification using a convolutional neural network (CNN) and achieved 92.1% accuracy (24). Later, Madani et al. used a CNN to classify 15 standard views, based on labeled still images and videos from 267 transthoracic echocardiograms that captured a range of real-world clinical variation (25). To our knowledge, there are no studies on how well view classification works in real life. However, every clinician knows that there is a learning curve to learn to practice ultrasound, and every clinician has seen a foreshortened recording of an inexperienced user in clinical practice. Evaluation demonstrated an accuracy of 91.7% among 15 views. Cheema et al. studied guiding of beginners toward a technically correct image with a DL technique (EchoGPS™, Bay Labs) (26). In this study 28 users with no prior training in echocardiography were evaluated on their ability to obtain images of 10 routine echo views on a standardized subject after a 1.5-h familiarization with the software. The mean percent of auto-captures was 69% in physicians, 72% in advance practice providers, 83% in registered nurses, and 70% in certified medical assistants. All participants were able to use static and dynamic guidance to improve image quality while scanning. As a resume, AI solutions are well-suited for image view classification tasks and are increasingly availably. Automation of these tasks improves learning curve of students and has clinical impact due to the collection of higher quality images that improve interpretation.

### Automated Function Assessment

Left ventricular (LV) function assessment and quantification have been most often performed because of the clinical importance (19, 25, 27–32). Several studies have evaluated AI-driven echocardiography image analysis methods including automated contour-based segmentation, see Table 1. Asch et al. used commercial software (AutoEF, BayLabs) with NN to perform LV EF estimation automatically on a database of more than 50.000 echocardiographic studies, including multiple AP2CH and AP4CH views. The evaluation on a set of 99 patients shows that the method performs similar to measurements of cardiologists with more than 20 years of experience: *r* = 0.94, bias=1.4%, limits of agreement = ±13.4%, sensitivity 0.93, specificity 0.87 (33).

**Table 1:** Artificial intelligence for image analysis and quantification.

**Authors**	**Summary**	**Data**			**Performance**	
		**Acquisition**	**Datasets**	**Patients**	**Metric value**	**Compared against**
**Left ventricular function assessment and quantification**						
Asch et al.	Automated EF using ML to assess LV function and volumes	2D	1	> 50.000	*r* = 0.95	EA
Cannesson et al.	Automated EF using AI to assess LV function and volumes	2D	1	218	*r* = 0.96	EA
Hubert et al.	Automated diastolic function assessment	2D	1	50	AUC 0.91	OVS
Knackstedt et al.	Automated EF and strain using ML to assess LV function	2D	4	255	ICC 0.83	EA
Lancaster et al.	Automated diastolic function assessment	2D	1	866	Kappa 0.62	OVS
Medvedofsky et al.	Automated EF using ML to assess LV function and volumes	3D	6	180	*r* 0.94	EA
Rahmouni et al.	Automated EF using AI to assess LV function and volumes	2D	1	92	*r* = 0.64	EA
Sabovik et al.	Automated diastolic function assessment	2D	1	1,407	AUC 0.88	OVS
Tsang et al.	Automated EF using ML to assess LV function and volumes	3D	1	159	*r* 0.87–0.96	EA
**Disease classification**						
Calleja et al.	Automated quantification using ML to assess aortic stenosis and regurgitation	3D	1	40	ICC 0.99	OIM
Casaclang et al.	Automated ventricular response to AS using ML	2D	1	246	*p* < 0.001	EA
Diller et al.	Automated segmentation using DL to detect congenital heart disease	2D	2	239	AUC 0.98	EA
Ghesu et al.	Automated detection valve morphology using DL	3D	X	869	CE 45.2%	CT
Jeganathan et al.	Evaluate valve morphology using AI in mitral valve analysis	3D	1	4	*P* = 0.0083	EA
Jin et al.	Automated localizing prolapse using ML to evaluate mitral insufficiency	3D	1	90	AC 0.89	EA
Madani et al.	Automated diagnosis ventricular hypertrophy using DL	2D	1	79.937	AUC 91.2	EA
Moghaddasi et al.	Automated quantification mitral regurgitation using ML	2D	1	102	AUC 0.99	EA
Narula et al.	Automated discrimination HCM or athlete heart using ML	2D	1	139	S&S *p* = 0.04	EA
Pereira et al.	Automated detection aortic coarctation using DL	2D	1	163	ER 12.9	EA
Sanchez et al.	Automated clustering using ML for group classification	2D	4	156	κ, 72.6%	EA
Sengupta et al.	Automated discrimination pericarditis or RCM using ML	2D	2	94	AUC 0.89	OIM
Zhang et al.	Automated discrimination HCM, amyloidosis, or PAH using DL	2D	1	14.035	AUC >0.84	EA

*AC, accuracy; AUC, area under curve; DC, dice coefficient; EA, expert assessment; EF, ejection fraction; EV, echo vendor; HCM, hypertrophic cardiomyopathy; IG, information gain; LV, left ventricular; ICC, intraclass correlation coefficient; OIM, other image modality; OVS, other validated scores; RCM, restrictive cardiomyopathy; SE, segmentation error; X, not available*.

Cannesson et al. performed an evaluation study using commercial software (AutoEF, Siemens) in 218 patients, including 165 patients with abnormal LV function (34). The AI solution was trained on more than 10,000 tracings by human experts to automatically locate and track the LV endocardium from routine grayscale digital loops and calculate EF. The AI solution correlated well with visual EF by expert readers (*r* = 0.96; *p* < 0.001) and performed analysis in 15 s per patient. However, less favorable results were found by Rahmouni et al. who evaluated the same AI algorithm and found discrepancies in EF estimates between AutoEF and manual tracing and between AutoEF and CMR (35). The authors recommended validation in a number of large, busy echocardiographic laboratories. Knackstedt et al. performed an external evaluation study using a commercial ML solution (AutoLV, TomTec) in 255 patients, of whom apical AP2CH and AP4CH views were collected from four centers that assessed EF using both visual estimation and manual tracing (27). ML was applied for calculating fully automated EF and longitudinal strain measurements. Interclass correlation coefficients and Bland-Altman analysis revealed good agreements among automated EF (ICC: 0.83, bias 0.7%, 95%), local center manual tracking, and reference center manual tracking, but not for visual EF assessments. 3D echocardiography, which can be obtained with more complex transducers, is increasingly available. In an evaluation study by Tsang et al. the commercial ML solution HeartModel was evaluated in 159 patients to quantify 3D echocardiography derived left atrial and LV volumes and LV EF (28). The AI technique strongly correlated with expert measurements (*r* = 0.87 to 0.96) and volumes and ejection fraction derived from magnetic resonance imaging (*r* = 0.84 to 0.95) using a 1.5-Tesla scanner (Achieva, Philips Healthcare). Medvedofsky et al. used the same ML tool on 3D echocardiographic images in 180 patients at six sites and demonstrated that LV EF and chamber volume were an accurate alternative to expert assessment (*r*: LVEDV: 0.99, LVESV: 0.99, LVEF: 0.94, LAV: 0.99) (36).

Evaluation of diastolic parameters is also important in assessing LV function of patients. Diastolic dysfunction is associated with increased myocardial fibrosis, increased ventricular stiffness and reduced prognosis (37, 38). Lancaster et al. used hierarchical clustering to discriminate between the different degrees of diastolic dysfunction, and improved prediction of event-free survival was found by clusters over conventional guideline-based classification for all-cause mortality and cardiac mortality (38). More recently, Hubert et al. reported on an AI solution for diastolic assessment in fifty patients (25 with amyloidosis, and 25 with heart failure with preserved ejection fraction) (39). This AI solution demonstrated a significant difference of the global area between both groups (37 vs. 72 mL%, respectively, *P* < 0.0001). Applying a linear discriminant analysis classifier, results showed a mean area under the curve (AUC) of 0.91 for the comparison between both groups. In this study classical indices of diastolic function were pathological in both groups with greater left atrial volume index, greater mitral average E/e' ratio, faster tricuspid regurgitation (*P* < 0.0001) compared to controls. Another study on AI and diastolic function was performed by Sabovčik et al. The authors applied an AI solution to detect early stages of cardiac remodeling and diastolic dysfunction in 1,407 participants (mean age, 51 years, 51% women) with an AUC curve with values between 86.2 and 88.1% (40). In conclusion, AI solutions might help to pre-select individuals in whom further echocardiographic examination, monitoring, and preventive measures are warranted.

The aforementioned studies show that AI solutions are increasingly developed for both systolic and diastolic LV function assessment and quantification. Use of these AI solutions is feasible. To conform the findings external evaluation and assessment of clinically relevant outcomes is required.

### Automated Disease Classification

Analysis of echocardiographic images plays a crucial role in clinical routine to measure the cardiac morphology to reach a diagnosis (41). Such analysis is based on the interpretation of clinical parameters which are extracted through image analysis such as segmentation and tracking. For instance, diagnosis of LV hypertrophy requires accurate delineation of the LV endocardium in both end diastole and end systole. As a next step labeled echocardiographic views from a patient with known pathology can be used to train an AI solution, or to automate disease classification in a new sample (42). In that case, the AI solution recognizes a pattern similar to what a technician or cardiologist recognizes.

#### Valvular Heart Disease

AI solutions are rapidly emerging for valvular heart disease, see Table 1 (12). AI can help with sizing and modeling of minimally invasive structural heart interventional devices, where possible with real-time guidance (43). Studies limited to internal validation demonstrate good performance. Moghaddasi et al. used supervised ML classifiers for assessment of mitral regurgitation (MR) severity in 102 patients with an accuracy of 99% (44). Ghesu et al. introduced Marginal Space DL to perform automated valve detection and segmentation in 869 patients with superior accuracy in corner error measured in millimeter as compared to cardiac computed tomography (45). ML was used to determine LV responses during the progression of aortic stenosis by Casaclang et al. and the authors demonstrated precise recognition of the pattern of LV responses during the progression of AS (*p* < 0.0001) (46). Another study was performed to determine the interobserver variability of automated 3D mitral valve analysis using commercial software (eSie Valve software, Siemens) (47). The authors found a high reproducibility in this study with a small data set size (*P* < 0.0083).

A number of studies focused on the evaluation of AI tools. Automated quantification of aortic stenosis and regurgitation in 3D trans esophageal echocardiography with ML was developed by a commercial vendor (Auto Valve, Siemens). External evaluation at Ohio State University showed an excellent performance as compared to expert assessment (ICC 0.99) (48). Jin et al. used ML to support both experts and non-experts in localizing mitral valve prolapse by 3D transesophageal echocardiography (49). The authors reported significantly improved accuracy of non-experts using the ML application (from 83 to 89%, *P* = 0.003). Moreover, significantly less time for image analysis was needed using ML by both experts (1.9 ± 0.7 vs. 9.9 ± 3.5 min, *P* < 0.0001) and non-experts (5.0 ± 0.5 vs. 13 ± 1.5 min, *P* < 0.0001), especially for complex pathology (49).

#### Cardiomyopathies

AI tools have potential to be used to discriminate various cardiomyopathies. Unfortunately, large multicenter studies are lacking and current studies are limited to evaluation utilizing single center data. For example, a supervised ML classifier was used to discriminate between restrictive cardiomyopathy and constrictive pericarditis using clinical and echocardiographic data of 50 patients with constrictive pericarditis and 44 with restrictive cardiomyopathy. The results demonstrated an excellent AUC of 96.2% (50). A similar study evaluated an ensemble combining three supervised classifiers (support vector machine, random forest, artificial neural network) to discriminate between hypertrophic cardiomyopathy and physiological hypertrophy in athletes (51). The results demonstrated superior performance compared to individual echocardiographic indices early-to-late diastolic transmitral velocity ratio, e', and strain (*p* = 0.04). In another work, unsupervised clustering approach was used to automatically classify 156 patients with a heart failure who underwent stress echocardiography. The method demonstrated good correlation with expert assessment (κ = 72.6%) (52). Later, CNN was successfully deployed by Zhang et al. to discriminate between diagnosis of hypertrophic cardiomyopathy, cardiac amyloidosis, and pulmonary arterial hypertension (AUC > 0.85) (42). Madani et al. utilized DL and clustering analysis of image classification in a small set of labeled data (4%) and a large set of unlabeled data for LV hypertrophy classification. The method achieved an accuracy of 0.92 (31). Other authors used CNNs to diagnose transposition of the great arteries or congenitally corrected transposition of the great arteries with 98.0% accuracy (29). Furthermore, a combination of autoencoder and support vector machine classifier was used to diagnose aortic coarctation using echocardiography data with high accuracy (53).

### Quality Assessment and Enhancement

Quality of echocardiograms is operator dependent, and can vary across patients and medical equipment. Patient characteristics such as fat, bone and air, breathing and patient movements may lead to reduced quality of clips and images and artifacts. AI can help technicians and cardiologists to support acquisitions and automate quality assessment and enhancement of echocardiograms (19, 54). Wu et al. demonstrated superiority of ML assisted echocardiogram enhancement over other image despeckling methods and video denoising methods as visually evaluated by experts (54). Abdi et al. performed a DL study in which 6,916 echo images were annotated by an expert on a five-point scale with a score between one (not acceptable) and five (excellent) (55). Internal evaluation demonstrated satisfactory accuracy (mean absolute error = 0.71) and <10 milliseconds computation time per frame, sufficient for real-time deployment. In summary, data on AI solutions for quality assessment and enhancement are limited but promising.

### Event Prediction

Risk assessment and prediction of both survival and cardiac events are key tasks in management of cardiac patients (56, 57). Studies on AI and event prediction in the field of echocardiography are listed in Table 2. In a study on in 866 patients referred for echocardiographic assessment automated prediction of major adverse cardiovascular events (MACE) was performed with ML cluster analysis (38). This technique was superior to conventional prediction techniques (AIC 157, kappa = 0.619, *p* < 0.001). Berchialla et al. used stress echocardiography data integrated with LV functional and angiographic data to predict MACE (58). The authors demonstrated discrimination ability superior or comparable of a Bayesian network to other ML classifiers. Ghorbani et al. developed a DL solution (EchoNet) to predict cardiovascular risk in 2,850 patients (59). Internal evaluation demonstrated a high accuracy of the DL solution to detect systemic factors such as age and sex from echocardiogram images alone (AUC 0.88), which is impossible for human experts. Kwon et al. focuses on the echocardiography reports and developed a DL solution using deep neural networks with TensorFlow (the Google Brain Team, Mountain View, United States) as the backend to predict survival from these reports in a multicenter retrospective cohort study on 25,776 patients with 1,026 mortalities (60). Importantly, the authors used derivation data of hospital A and performed external evaluation using echocardiography reports of hospital B. The authors obtained superior performance as compared to conventional prediction models (AUC = 0.88). In another study that focused on echocardiography reports Samad et al. predicted survival in 171,510 unselected patients who underwent 331,317 echocardiograms (61). The authors achieved a significantly higher prediction accuracy with nonlinear ML over linear logistic regression models (AUC>0.82). A model including clinical variables, LV function and 57 echocardiographic measurements yielded the highest prediction accuracy (*p* < 0.01 across all models and survival durations). In conclusion, AI solutions for risk assessment and prediction of both survival and cardiac events are promising, but require further evaluation. Most data are obtained from retrospective studies and evaluation on external datasets is often lacking.

**Table 2:** Artificial intelligence solutions for prediction of events.

**For Authors**	**Summary**	**Data**				**Performance**	
		**Acquisition**	**Datasets**	**Patients**	**Follow-up (m)**	**Metric value**	**Compared against**
**Cardiovascular risk prediction**							
Ghorbani et al.	Automated cardiovascular risk prediction using DL	2D	1	2.850	0	AUC 0.88	EA
Survival prediction
Kwon et al.	Automated risk prediction using DL	2D	2	4.759	36	AUC 0.88	OVS
Samad et al.	Automated risk prediction using ML	2D	1	171.510	60	AUC >0.82	OVS
**Adverse events prediction**							
Berchialla et al.	Automated event prediction using ML	2D	1	228	X	PoV 0.70	ML
Lancaster et al.	Automated event prediction using ML	2D	1	866	48	AIC 157	OVS

*AIC, Akaike information criterion; AUC, area under the curve; EA, expert assessment; HR, hazard ratio; m, months; OCV, optimal cutoff value; OVS, other validated scores; PoV, part of variance; N/A, not applicable; X, not available*.

## Discussion

We summarize eleven studies on the development of AI in the field of routine echocardiography. Overall performance of the AI solutions was comparable to expert performance. However, these studies were virtually all hampered by lack of external validation of multi-center datasets. Eight studies evaluated performance of commercial software, all for diagnostic purposes.

To this end most successful AI methods are supervised, in other words they learn from labeled data. Hence, performance of supervised AI solutions depends on careful labeling of input data, which often relies on the technicians and cardiologists. Intra- and interobserver variability in data labeling may limit AI performance (30, 33). The availability of large, diverse and labeled data is a prerequisite for progress in the development and evaluation of AI solutions. Last year two large open datasets in the field of 2D echocardiography became publicly available; from Stanford University and University of Lyon (32, 41). This opens exciting possibilities. Nevertheless, the data are limited to echocardiography videos only and not accompanied by relevant clinical patient data and outcomes. Another challenge has been the lack of data standardization (62). Poor data standardization leads to incomplete and inaccurate data collection, patient matching issues, and slower workflows. Large sets with reference labels and standardized evaluation procedures would allow better comparison between the methods. An itemized checklist that highlights steps for ensuring correct application of AI models and the consistent reporting of model specifications and result might help (63). Another challenge are potential ethical problems derived from data sharing or de-identification to maintain patients' privacy. Consequently, these studies need additional local institutional review board authorizations to evaluate appropriate use of data.

In the future it is to be expected that AI solutions will increasingly support technicians and cardiologists in the field of digital care (64, 65) and echocardiography (66). AI can be incorporated into everyday practice and become a valuable aid for cardiologists and technicians dealing with cardiovascular disease (67, 68). AI will help to reduce workload, increase reproducibility and standardize data reporting. AI is also expected to improve study preparation by all related views retrieved automatically. This would save the technician or cardiologist time in searching through the complete study with sometimes hundreds of images by allowing the them to visualize all requested information quickly. AI is expected to improve echocardiography acquisition with support on automated probe adjustments and recording leading to advances in efficiency and overcome human limitations of both distraction and fatigue. Automated acquisitions will additionally contribute to increased standardization. Future AI solutions are also expected to extract information not directly apparent to humans (43). Improved prediction of events and mortality is expected with new data driven AI solutions, preferably in real time (43). On an educational level, much more can be expected from automated disease classification. As a beginner it can be difficult to distinguish between normal and abnormal structures, and with AI support that may become much easier. So far, most educational studies focused on automated view classification to recognize off-axis acquisition and incorrect views and provide guidance on how to move the probe in order to obtain diagnostic images.

Specifically, for echocardiography, there is a major challenge in the absence of standardization of the image sets and the varying image quality. Datasets with CT and MRI images are often obtained in a more standardized manner, but this researcher has been performed in fewer patients. Echocardiography is one of the basic researches in cardiology, and therefore challenge in image quality are certainly not insurmountable because of the extensive data volume.

As a resume, contemporary AI solutions for image acquisition, analysis, reporting and education are developed for relevant tasks with promising performance. Studies with external validation must show whether successful performance is sustained. In the future major benefit of AI in echocardiography is expected from improvements in automated interpretation and event prediction to reduce workload and improve clinical outcome. Some of the discussed challenges have yet to be overcome, however, none of them are insurmountable. Studies are also needed to acquire trust in new technologies, supported by efforts toward explainable models and transparency.

## Author Contributions

MS, II, and BB drafted the manuscript, which was critically revised and edited by BC and SC. All authors agree to be accountable for all aspects of the work.

## Conflict of Interest

II is cofounder and Scientific Lead of Quantib-U BV. The remaining authors declare that the research was conducted in the absence of any commercial or financial relationships that could be construed as a potential conflict of interest.

## References

[1] Knuuti J Wijns W Saraste A Capodanno D Barbato E Funck-Brentano C. 2019 ESC Guidelines for the diagnosis and management of chronic coronary syndromes. Eur Heart J. (2020) 41:407–77. 10.1093/eurheartj/ehz425 31504439

[2] Baumgartner H Falk V Bax JJ De Bonis M Hamm C Holm PJ. 2017 ESC/EACTS Guidelines for the management of valvular heart disease. Eur Heart J. (2017) 38:2739–91. 10.5603/KP.2018.0013 28886619

[3] Steeds RP Garbi M Cardim N Kasprzak JD Sade E Nihoyannopoulos P. EACVI appropriateness criteria for the use of transthoracic echocardiography in adults: a report of literature and current practice review. Eur Heart J Cardiovasc Imaging. (2017) 18:1191–204. 10.1093/ehjci/jew333 28329307

[4] Bouma BJ Riezenbos R Voogel AJ Veldhorst MH Jaarsma W Hrudova J. Appropriate use criteria for echocardiography in the Netherlands. Neth Heart J. (2017) 25:330–4. 10.1007/s12471-017-0960-9 28247246 PMC5405027

[5] Sengupta PP Adjeroh DA. Will artificial intelligence replace the human echocardiographer? Circulation. (2018) 138:1639–42. 10.1161/CIRCULATIONAHA.118.037095 30354473 PMC6448766

[6] Galderisi M Cosyns B Edvardsen T Cardim N Delgado V Di Salvo G. Standardization of adult transthoracic echocardiography reporting in agreement with recent chamber quantification, diastolic function, and heart valve disease recommendations: an expert consensus document of the European Association of Cardiovascular Imaging. Eur Heart J Cardiovasc Imaging. (2017) 18:1301–10. 10.1093/ehjci/jex244 29045589

[7] Klem I Shah DJ White RD Pennell DJ van Rossum AC Regenfus M. Prognostic value of routine cardiac magnetic resonance assessment of left ventricular ejection fraction and myocardial damage: an international, multicenter study. Circ Cardiovasc Imaging. (2011) 4:610–9. 10.1161/CIRCIMAGING.111.964965 21911738

[8] Kusunose K Haga A Abe T Sata M. Utilization of artificial intelligence in echocardiography. Circ J. (2019) 83:1623–9. 10.1253/circj.CJ-19-0420 31257314

[9] Krittanawong C Johnson KW Rosenson RS Wang Z Aydar M Baber U. Deep learning for cardiovascular medicine: a practical primer. Eur Heart J. (2019) 40:2058–73. 10.1093/eurheartj/ehz056 30815669 PMC6600129

[10] Mitchell C Rahko PS Blauwet LA Canaday B Finstuen JA Foster MC. Guidelines for performing a comprehensive transthoracic echocardiographic examination in adults: recommendations from the American Society of Echocardiography. J Am Soc Echocardiogr. (2019) 32:1–64. 10.1016/j.echo.2018.06.004 30282592

[11] Ranschaert E Morozov S Algra P. Artificial Intelligence in Medical Imaging: Opportunities, Applications and Risks. Cham: Springer Nature Switzerland AG (2019). 10.1007/978-3-319-94878-2

[12] Davis A Billick K Horton K Jankowski M Knoll P Marshall JE. Artificial intelligence and echocardiography: a primer for cardiac sonographers. J Am Soc Echocardiogr. (2020) 33:1061–6. 10.1016/j.echo.2020.04.025 32536431 PMC7289098

[13] Omar AMS Krittanawong C Narula S Narula J Argulian E. Echocardiographic data in artificial intelligence research: primer on concepts of big data and latent states. JACC Cardiovasc Imaging. (2020) 13:170–2. 10.1016/j.jcmg.2019.07.017 31542543

[14] Siegersma KR Leiner T Chew DP Appelman Y Hofstra L Verjans JW. Artificial intelligence in cardiovascular imaging: state of the art and implications for the imaging cardiologist. Neth Heart J. (2019) 27:403–13. 10.1007/s12471-019-01311-1 31399886 PMC6712136

[15] Benjamins JW Hendriks T Knuuti J Juarez-Orozco LE van der Harst P. A primer in artificial intelligence in cardiovascular medicine. Neth Heart J. (2019) 27:392–402. 10.1007/s12471-019-1286-6 31111458 PMC6712147

[16] Seetharam K Brito D Farjo PD Sengupta PP. The role of artificial intelligence in cardiovascular imaging: state of the art review. Front Cardiovasc Med. (2020) 7:618849. 10.3389/fcvm.2020.618849 33426010 PMC7786371

[17] Deo RC. Machine learning in medicine. Circulation. (2015) 132:1920–30. 10.1161/CIRCULATIONAHA.115.001593 26572668 PMC5831252

[18] LeCun Y Bengio Y Hinton G. Deep learning. Nature. (2015) 521:436–44. 10.1038/nature14539 26017442

[19] Al'Aref SJ Anchouche K Singh G Slomka PJ Kolli KK Kumar A. Clinical applications of machine learning in cardiovascular disease and its relevance to cardiac imaging. Eur Heart J. (2019) 40:1975–86. 10.1093/eurheartj/ehy404 30060039

[20] Wallis C. How artificial intelligence will change medicine. Nature. (2019) 576:S48. 10.1038/d41586-019-03845-1 31853072

[21] Litjens G Ciompi F Wolterink JM de Vos BD Leiner T Teuwen J. State-of-the-art deep learning in cardiovascular image analysis. JACC Cardiovasc Imaging. (2019) 12:1549–65. 10.1016/j.jcmg.2019.06.009 31395244

[22] Slomka PJ Dey D Sitek A Motwani M Berman DS Germano G. Cardiac imaging: working towards fully-automated machine analysis & interpretation. Expert Rev Med Devices. (2017) 14:197–212. 10.1080/17434440.2017.1300057 28277804 PMC5450918

[23] Khamis H Zurakhov G Azar V Raz A Friedman Z Adam D. Automatic apical view classification of echocardiograms using a discriminative learning dictionary. Med Image Anal. (2017) 36:15–21. 10.1016/j.media.2016.10.007 27816858

[24] Gao X Li W Loomes M Wang L. A fused deep learning architecture for viewpoint classification of echocardiography. Information Fusion. (2017) 36:103–113. 10.1016/j.inffus.2016.11.007

[25] Madani A Arnaout R Mofrad M Arnaout R. Fast and accurate view classification of echocardiograms using deep learning. NPJ Digit Med. (2018). 10.1038/s41746-017-0013-1. [Epub ahead of print]. 30828647 PMC6395045

[26] Baljash C Ching H David A Akhil N James DT. Abstract 15694: automated guidance and image capture of echocardiographic views using a deep learning-derived technology. Circulation. (2019) 140:A15694. 10.1161/circ.140.suppl_1.15694

[27] Knackstedt C Bekkers SCAM Schummers G Schreckenberg M Muraru D Badano LP. Fully automated versus standard tracking of left ventricular ejection fraction and longitudinal strain: the FAST-EFs multicenter study. J Am Coll Cardiol. (2015) 66:1456–66. 10.1016/j.jacc.2015.07.052 26403342

[28] Tsang W Salgo IS Medvedofsky D Takeuchi M Prater D Weinert L. Transthoracic 3D echocardiographic left heart chamber quantification using an automated adaptive analytics algorithm. JACC Cardiovasc Imaging. (2016) 9:769–82. 10.1016/j.jcmg.2015.12.020 27318718

[29] Diller G-P Babu-Narayan S Li W Radojevic J Kempny A Uebing A. Utility of machine learning algorithms in assessing patients with a systemic right ventricle. Eur Heart J Cardiovasc Imaging. (2019) 20:925–31. 10.1093/ehjci/jey211 30629127 PMC6639730

[30] Gandhi S Mosleh W Shen J Chow CM. Automation, machine learning, and artificial intelligence in echocardiography: a brave new world. Echocardiography. (2018) 35:1402–18. 10.1111/echo.14086 29974498

[31] Madani A Ong JR Tibrewal A Mofrad MRK. Deep echocardiography: data-efficient supervised and semi-supervised deep learning towards automated diagnosis of cardiac disease. NPJ Digit Med. (2018) 1:59. 10.1038/s41746-018-0065-x 31304338 PMC6550282

[32] Ouyang D He B Ghorbani A Yuan N Ebinger J Langlotz CP. Video-based AI for beat-to-beat assessment of cardiac function. Nature. (2020) 580:252–6. 10.1038/s41586-020-2145-8 32269341 PMC8979576

[33] Asch FM Poilvert N Abraham T Jankowski M Cleve J Adams M. Automated echocardiographic quantification of left ventricular ejection fraction without volume measurements using a machine learning algorithm mimicking a human expert. Circ Cardiovasc Imaging. (2019) 12:e009303. 10.1161/CIRCIMAGING.119.009303 31522550 PMC7099856

[34] Cannesson M Tanabe M Suffoletto MS McNamara DM Madan S Lacomis JM. A novel two-dimensional echocardiographic image analysis system using artificial intelligence-learned pattern recognition for rapid automated ejection fraction. J Am Coll Cardiol. (2007) 49:217–26. 10.1016/j.jacc.2006.08.045 17222733

[35] Rahmouni HW Ky B Plappert T Duffy K Wiegers SE Ferrari VA. Clinical utility of automated assessment of left ventricular ejection fraction using artificial intelligence-assisted border detection. Am Heart J. (2008) 155:562–70. 10.1016/j.ahj.2007.11.002 18294497

[36] Medvedofsky D Mor-Avi V Amzulescu M Fernández-Golfín C Hinojar R Monaghan MJ. Three-dimensional echocardiographic quantification of the left-heart chambers using an automated adaptive analytics algorithm: multicentre validation study. Eur Heart J Cardiovasc Imaging. (2018) 19:47–58. 10.1093/ehjci/jew328 28159984

[37] Importance of Diastolic Function for the Prediction of Arrhythmic Death | Circulation: Arrhythmia and Electrophysiology. Available online at: https://www.ahajournals.org/10.1161/CIRCEP.119.007757 PMC7141079 31944144

[38] Lancaster MC Salem Omar AM Narula S Kulkarni H Narula J Sengupta PP. Phenotypic clustering of left ventricular diastolic function parameters: patterns and prognostic relevance. JACC Cardiovasc Imaging. (2019) 12:1149–61. 10.1016/j.jcmg.2018.02.005 29680357

[39] Hubert A Le Rolle V Galli E Bidaud A Hernandez A Donal E. New expectations for diastolic function assessment in transthoracic echocardiography based on a semi-automated computing of strain-volume loops. Eur Heart J Cardiovasc Imaging. (2020) 21:1366–71. 10.1093/ehjci/jeaa123 33245757

[40] Sabovčik F Cauwenberghs N Kouznetsov D Haddad F Alonso-Betanzos A Vens C. Applying machine learning to detect early stages of cardiac remodelling and dysfunction. Eur Heart J Cardiovasc Imaging. (2020). 10.1093/ehjci/jeaa135. [Epub ahead of print]. 32588036

[41] Leclerc S Smistad E Pedrosa J Ostvik A Cervenansky F Espinosa F. Deep Learning for Segmentation Using an Open Large-Scale Dataset in 2D Echocardiography. IEEE Trans Med Imaging. (2019) 38:2198–2210. 10.1109/TMI.2019.2900516 30802851

[42] Zhang J Gajjala S Agrawal P Tison GH Hallock LA Beussink-Nelson L. Fully automated echocardiogram interpretation in clinical practice. Circulation. (2018) 138:1623–35. 10.1161/CIRCULATIONAHA.118.034338 30354459 PMC6200386

[43] Alsharqi M Woodward WJ Mumith JA Markham DC Upton R Leeson P. Artificial intelligence and echocardiography. Echo Res Pract. (2018) 5:R115–25. 10.1530/ERP-18-0056 30400053 PMC6280250

[44] Moghaddasi H Nourian S. Automatic assessment of mitral regurgitation severity based on extensive textural features on 2D echocardiography videos. Comput Biol Med. (2016) 73:47–55. 10.1016/j.compbiomed.2016.03.026 27082766

[45] Ghesu FC Krubasik E Georgescu B Singh V Yefeng Zheng null Hornegger J. Marginal space deep learning: efficient architecture for volumetric image parsing. IEEE Trans Med Imaging. (2016) 35:1217–28. 10.1109/TMI.2016.2538802 27046846

[46] Casaclang-Verzosa G Shrestha S Khalil MJ Cho JS Tokodi M Balla S. Network tomography for understanding phenotypic presentations in aortic stenosis. JACC Cardiovasc Imaging. (2019) 12:236–48. 10.1016/j.jcmg.2018.11.025 30732719

[47] Jeganathan J Knio Z Amador Y Hai T Khamooshian A Matyal R. Artificial intelligence in mitral valve analysis. Ann Card Anaesth. (2017) 20:129–34. 10.4103/aca.ACA_243_16 28393769 PMC5408514

[48] Calleja A Thavendiranathan P Ionasec RI Houle H Liu S Voigt I. Automated quantitative 3-dimensional modeling of the aortic valve and root by 3-dimensional transesophageal echocardiography in normals, aortic regurgitation, and aortic stenosis: comparison to computed tomography in normals and clinical implications. Circ Cardiovasc Imaging. (2013) 6:99–108. 10.1161/CIRCIMAGING.112.976993 23233743

[49] Jin C-N Salgo IS Schneider RJ Kam KK-H Chi W-K So C-Y. Using anatomic intelligence to localize mitral valve prolapse on three-dimensional echocardiography. J Am Soc Echocardiogr. (2016) 29:938–45. 10.1016/j.echo.2016.07.002 27545445

[50] Sengupta PP Huang Y-M Bansal M Ashrafi A Fisher M Shameer K. Cognitive machine-learning algorithm for cardiac imaging: a pilot study for differentiating constrictive pericarditis from restrictive cardiomyopathy. Circ Cardiovasc Imaging. (2016) 9:e004330. 10.1161/CIRCIMAGING.115.004330 27266599 PMC5321667

[51] Narula S Shameer K Salem Omar AM Dudley JT Sengupta PP. Machine-learning algorithms to automate morphological and functional assessments in 2D echocardiography. J Am Coll Cardiol. (2016) 68:2287–95. 10.1016/j.jacc.2016.08.062 27884247

[52] Sanchez-Martinez S Duchateau N Erdei T Kunszt G Aakhus S Degiovanni A. Machine learning analysis of left ventricular function to characterize heart failure with preserved ejection fraction. Circ Cardiovasc Imaging. (2018) 11:e007138. 10.1161/CIRCIMAGING.117.007138 29661795

[53] Pereira F Bueno A Rodriguez A Perrin D Marx G Cardinale M. Automated detection of coarctation of aorta in neonates from two-dimensional echocardiograms. J Med Imaging (Bellingham). (2017) 4:014502. 10.1117/1.JMI.4.1.014502 28149925 PMC5260631

[54] Wu H Huynh TT Souvenir R. Echocardiogram enhancement using supervised manifold denoising. Med Image Anal. (2015) 24:41–51. 10.1016/j.media.2015.05.004 26072166

[55] Abdi AH Luong C Tsang T Allan G Nouranian S Jue J. Automatic quality assessment of echocardiograms using convolutional neural networks: feasibility on the apical four-chamber view. IEEE Trans Med Imaging. (2017) 36:1221–30. 10.1109/TMI.2017.2690836 28391191

[56] Schuuring MJ Vis JC van Dijk APJ van Melle JP Vliegen HW Pieper PG. Impact of bosentan on exercise capacity in adults after the Fontan procedure: a randomized controlled trial. Eur J Heart Fail. (2013) 15:690–8. 10.1093/eurjhf/hft017 23361871

[57] Schuuring MJ van Riel ACMJ Vis JC Duffels MG van Dijk APJ de Bruin-Bon RHACM. New predictors of mortality in adults with congenital heart disease and pulmonary hypertension: midterm outcome of a prospective study. Int J Cardiol. (2015) 181:270–6. 10.1016/j.ijcard.2014.11.222 25535690

[58] Berchialla P Foltran F Bigi R Gregori D. Integrating stress-related ventricular functional and angiographic data in preventive cardiology: a unified approach implementing a Bayesian network. J Eval Clin Pract. (2012) 18:637–43. 10.1111/j.1365-2753.2011.01651.x 21449973

[59] Ghorbani A Ouyang D Abid A He B Chen JH Harrington RA. Deep learning interpretation of echocardiograms. NPJ Digit Med. (2020) 3:10. 10.1038/s41746-019-0216-8 31993508 PMC6981156

[60] Kwon JM Kim KH Jeon KH Park J. Deep learning for predicting in-hospital mortality among heart disease patients based on echocardiography. Echocardiography. (2019) 36:213–8. 10.1111/echo.14220 30515886

[61] Samad MD Ulloa A Wehner GJ Jing L Hartzel D Good CW. Predicting survival from large echocardiography and electronic health record datasets: optimization with machine learning. JACC Cardiovasc Imaging. (2019) 12:681–9. 10.1016/j.jcmg.2018.04.026 29909114 PMC6286869

[62] Badano LP Kolias TJ Muraru D Abraham TP Aurigemma G Edvardsen T. Standardization of left atrial, right ventricular, and right atrial deformation imaging using two-dimensional speckle tracking echocardiography: a consensus document of the EACVI/ASE/Industry Task Force to standardize deformation imaging. Eur Heart J Cardiovasc Imaging. (2018) 19:591–600. 10.1093/ehjci/jey042 29596561

[63] Sengupta PP Shrestha S Berthon B Messas E Donal E Tison GH. Proposed requirements for cardiovascular imaging-related machine learning evaluation (PRIME): a checklist: reviewed by the american college of cardiology healthcare innovation council. JACC Cardiovasc Imaging. (2020) 13:2017–35. 10.1016/j.jcmg.2020.07.015 32912474 PMC7953597

[64] Schuuring MJ Kauw D Bouma BJ. COVID-19 pandemic: practical considerations on rapid initiation of remote care in chronic cardiac patients. Eur Heart J Digital Health. (2020) 1:8–9. 10.1093/ehjdh/ztaa007 PMC7717188 34192273

[65] Schuuring MJ Kauw D. How to initiate eHealth in congenital heart disease patients? Eur Heart J Digital Health. (2020) 1:83–6. 10.1093/ehjdh/ztaa012 PMC9707933 36713962

[66] Lang RM Addetia K Miyoshi T Kebed K Blitz A Schreckenberg M. Use of machine learning to improve echocardiographic image interpretation workflow: a disruptive paradigm change? J Am Soc Echocardiogr. (2020). 10.1016/j.echo.2020.11.017. [Epub ahead of print]. 33276079 PMC8026622

[67] Kusunose K. Steps to use artificial intelligence in echocardiography. J Echocardiogr. (2020). 10.1007/s12574-020-00496-4. [Epub ahead of print]. 33044715 PMC7549428

[68] Seetharam K Raina S Sengupta PP. The role of artificial intelligence in echocardiography. Curr Cardiol Rep. (2020) 22:99. 10.1007/s11886-020-01329-7 32728829

